# The association between heparin sensitivity index and postoperative blood loss in Chinese patients undergoing elective off-pump coronary artery bypass grafting: a single center retrospective study

**DOI:** 10.1186/s13019-024-02966-7

**Published:** 2024-07-08

**Authors:** Bin Jia, Chenying Ding, Jinhe Deng, Wenhui Qi, Yuntai Yao, The Evidence in Cardiovascular Anesthesia Group

**Affiliations:** 1 Department of Anesthesiology, The Second Affiliated Hospital of Guangzhou University of Chinese Medicine, Guangzhou, 510006 China; 2 Department of Anesthesiology, Fuwai Hospital, National Center for Cardiovascular Diseases, Chinese Academy of Medical Sciences and Peking Union Medical College, No. 167, Beilishi Road, Xicheng District, Beijing, 100037 China; 3 Department of Anesthesiology, The First Hospital of Hohhot, Hohhot, 010020 China; 4 Department of Anesthesiology, Harrison International Peace Hospital, Hengshui, 053000 China

**Keywords:** Heparin sensitivity index, Coronary artery bypass, Blood loss, Platelet count

## Abstract

**Background:**

The heparin sensitivity index (HSI) is closely associated with perioperative ischemic events and increased blood loss in cardiac surgery. Previous studies have produced conflicting results. Therefore, this study aimed to investigate the relationship between HSI and postoperative blood loss specifically in Chinese patients undergoing elective off-pump coronary artery bypass grafting (OPCAB).

**Methods:**

Patients underwent OPCAB between March 2021 and July 2022 were retrospectively included. Enrolled patients were classified into Low-HSI (HSI_LOW_; HSI < 1.3) and Normal-HSI (HSI_NORM_; HSI ≥ 1.3) groups. HSI = [(activated clotting time (ACT) after heparin) – (baseline ACT)] / [loading dose of heparin (IU/kg)]. Primary outcome included postoperative blood loss at 24 h. Secondary outcomes were total postoperative blood loss, transfusion requirement of red blood cell (RBC), fresh frozen plasma (FFP), platelet concentrates (PC), and other complications.

**Results:**

We retrospectively analyzed 303 Chinese OPCAB patients. HSI_LOW_ group had higher preoperative platelet (PLT) count (221 × 10^9^/L *vs*. 202 × 10^9^/L; *P* = 0.041) and platelet crit (PCT) value (0.23% *vs*. 0.22%; *P* = 0.040) compared to HSI_NORM_ group. Two groups showed no significant differences in postoperative blood loss at 24 h (460 mL *vs.* 470 mL; *P* = 0.252), total blood loss (920 mL *vs.* 980 mL; *P* = 0.063), RBC transfusion requirement (3.4% *vs.* 3.1%; *P* = 1.000), FFP transfusion requirement (3.4% *vs.* 6.2%; *P* = 0.380), and other complications. Preoperative high PLT count was associated with low intraoperative HSI value (odds ratio: 1.006; 95% confidence interval: 1.002, 1.011; *P* = 0.008).

**Conclusions:**

Intraoperative HSI value was not associated with postoperative blood loss in Chinese patients undergoing OPCAB. Preoperative high PLT count was an independent predictor of low intraoperative HSI value.

## Introduction

Unfractionated heparin (UFH) is widely used as an anticoagulant to prevent thromboembolic complications in cardiac surgery. However, the complex pharmacological properties of UFH may result in significant inter-patient variability in terms of responsiveness [1]. Various criteria are available for assessing heparin responsiveness: the general definition is whether UFH loading dose ranging from 150 IU/kg to 600 IU/kg can achieve a target activated clotting time (ACT) value of at least 300 s–600 s [2, 3]. To accommodate different UFH administration practices, some scholars have proposed using heparin sensitivity index (HSI) value to indicate differences in heparin responsiveness [4–6]. HSI is calculated using the following equation [5, 7].

$$\text{HSI}=\frac{\text{postheparin ACT }\left(\text{s}\right)-\text{ baseline ACT}(\text{s})}{\text{loading dose of heparin }(\text{IU}/\text{kg})}$$

Previous studies have shown that low HSI value are associated with increased postoperative blood loss in cardiac surgery [8, 9]. However, these studies only enrolled patients undergoing on-pump cardiac surgery. The relationship between interoperative HSI and postoperative blood loss in patients undergoing off-pump coronary artery bypass grafting (OPCAB) remains unknown.

Therefore, this study aimed to investigate the relationship between interoperative HSI and postoperative blood loss in patients undergoing OPCAB to provide a basis for individualized anticoagulation.

## Methods

### Ethics approval

This study was approved by the ethics committee of Fuwai Hospital (20211636). The need for obtaining informed patient consent was waived due to the retrospective nature of this study.

### Study population and design

Consecutive patients who underwent elective OPCAB at Fuwai Hospital between March 2021 and July 2022 were retrospectively enrolled. The inclusion criteria were as follows: (1) adult patients (> 18 years of age) and (2) patients undergoing OPCAB. Exclusion criteria were as follows: (1) emergency surgery, (2) previous cardiac surgery, (3) OPCAB combined with other surgery, (4) use of an intra-aortic balloon pump (IABP) prior to surgery, (5) a history of heparin-induced thrombocytopenia, (6) abnormal preoperative coagulation, thrombotic, or hematological diseases, (7) renal failure (serum creatinine > 200 mmol/L or urine output < 10 mL/h), (8) liver dysfunction (alanine aminotransferase, aspartate aminotransferase, alkaline phosphatase, or total bilirubin levels two times higher than the upper limit of normal), and (9) infectious endocarditis.

### Anesthetic and surgical protocols

General anesthesia was induced with midazolam 0.1 mg/kg, etomidate 2 mg/kg, sufentanil 2 μg/kg–3 μg/kg, and cis-atracurium 0.15 mg/kg–0.2 mg/kg. Propofol 100 mg/h–200 mg/h, dexmedetomidine 30 μg/h–50 μg/h, cis-atracurium 8 mg/h–15 mg/h, and sevoflurane 0.5%–1.5% were administered for anesthesia maintenance. Median sternotomy was performed on all patients. Bispectral index (BIS) was maintained between 40 and 60 during surgery. All patients received intraoperative autologous blood salvage using cell-saver systems. No patient received tranexamic acid. The patients were transferred to the intensive care unit (ICU) for postoperative care, and aspirin and clopidogrel were administered on the first postoperative day.

### Heparin sensitivity index

Patients underwent radial artery catheterization under local anesthesia prior to general anesthesia, and a 0.5 mL blood sample was collected from each patient's radial artery to determine baseline ACT. The Hemochron Signature Elite Response Whole Blood Coagulation System (International Technidyne Corporation (ITC), San Diego and Edison, USA) was used to monitor ACT using a Hemochron ACT^+^ cartridge (kaolin and silica activators). With a UFH loading dose of 200 IU/kg was used to achieve an activated ACT value of at least 300 s. Five minutes after UFH administration, a blood sample (0.5 mL) was collected from the radial artery to determine the post-heparin ACT. UFH 100 IU/kg was administered hourly following the initial dose, with the maintenance of an intraoperative ACT value ≥ 300 s [10]. UFH was neutralized with 2 mg/kg protamine after the conclusion of revascularization. ACT was subsequently measured 10 min after protamine neutralization. The criterion used to indicate reduced heparin responsiveness was HSI value < 1.3 [7]. HSI value ≥ 1.3 was considered to indicate normal heparin responsiveness. Enrolled patients were classified into two groups, namely Low-HSI (HSI_LOW_; HSI < 1.3) and Normal-HSI (HSI_NORM_; HSI ≥ 1.3) groups.

### Primary and secondary outcomes

The primary outcome was postoperative blood loss at 24 h. The secondary outcomes included total postoperative blood loss, transfusion requirement of red blood cell (RBC), fresh frozen plasma (FFP), and platelet concentrates (PC), postoperative mechanical ventilation duration, chest drainage duration, length of stay (LOS) in the ICU and hospital, incidences of reoperation for blood loss, cardiac arrest, myocardial infarction (MI), use of IABP, atrial fibrillation (AF), pacemaker implantation, neurological events (stroke and transient ischemic attack), delirium, renal replacement therapy, and mortality.

### Criteria for allogeneic transfusions and outcomes measures

Blood products were transfused according to the following protocols: the transfusion threshold for RBC was hemoglobin < 8.0 g/L. The indication for FFP transfusion was diffuse blood loss with a prothrombin time value 1.5 times longer than the baseline value. The triggers for PC transfusion were a platelet (PLT) count of < 50 × 10^9^/L or PLT dysfunction. Postoperative blood loss was defined based on the chest drainage volume. The patient underwent reoperation if the postoperative chest drainage volume exceeded 300 mL/h within the first 2 h or remained above 200 mL/h for 4 h. Removal of the chest drainage tubes was only considered when discharge fell below 20 mL for a period of at least 5 h–6 h [10]. Antiplatelet therapy (aspirin and/or clopidogrel) was discontinued on admission. Antithrombotic prophylaxis consisted of subcutaneous administration of low-molecular-weight heparin (LMWH) from admission until 12 h prior to surgery. Preoperative use of clopidogrel was defined as cessation within five days, whereas preoperative use of aspirin was defined as cessation within six days [11]. The protamine/UFH dose ratio was determined using the following formula: 1 mg of protamine per 100 IU of UFH. Clinical diagnostic criteria for MI were defined as the occurrence of an increase in cardiac enzyme levels five times higher than the upper limit of normal, at the same time, it must satisfy at least one of the following conditions: newly developed ischemic electrocardiographic changes, new Q-waves formation on electrocardiogram, imaging evidence of new myocardial infarction or new localized abnormal ventricular wall motion. Cerebral stroke was defined as a novel focal or global cerebral dysfunction that persisted for more than 24 h.

### Statistical analysis

The previous study [8] showed that the weighted mean difference in postoperative blood loss at 24 h between patients with Low-HSI and Normal-HSI undergoing cardiac surgery was 95 ml, with a standard deviation of 218.5 ml. Based on an independent t-test, we calculated that a sample size of 226 patients would be required to achieve a power of 90% at a two-sided significance level of 0.05. Considering a potential dropout rate of 20%, the minimum sample size was 272 patients. Our final sample size was increased to include 303 patients.

Categorical variables were used the χ^*2*^ test and expressed as numbers and percentage. Continuous variables were used the Wilcoxon rank-sum test and expressed as median and interquartile range. Repeated measurements of data were analyzed using two-way repeated measures analysis of variance, with data presented as mean ± standard deviation. A multivariate logistic regression model was used to examine the clinical and laboratory predictors of HSI. Baseline variables that were considered clinically relevant or variables with *P* < 0.20 in the univariate analysis were entered into the multivariable logistic regression model. The variables for inclusion were carefully chosen, given the number of events available, to ensure parsimony in the final model. *P* < 0.05 was considered to indicate statistical significance. All statistical analyses were performed using SPSS software (SPSS version 23.0; IBM Corp., Armonk, USA).

## Results

### Study population

**Fig. 1:**
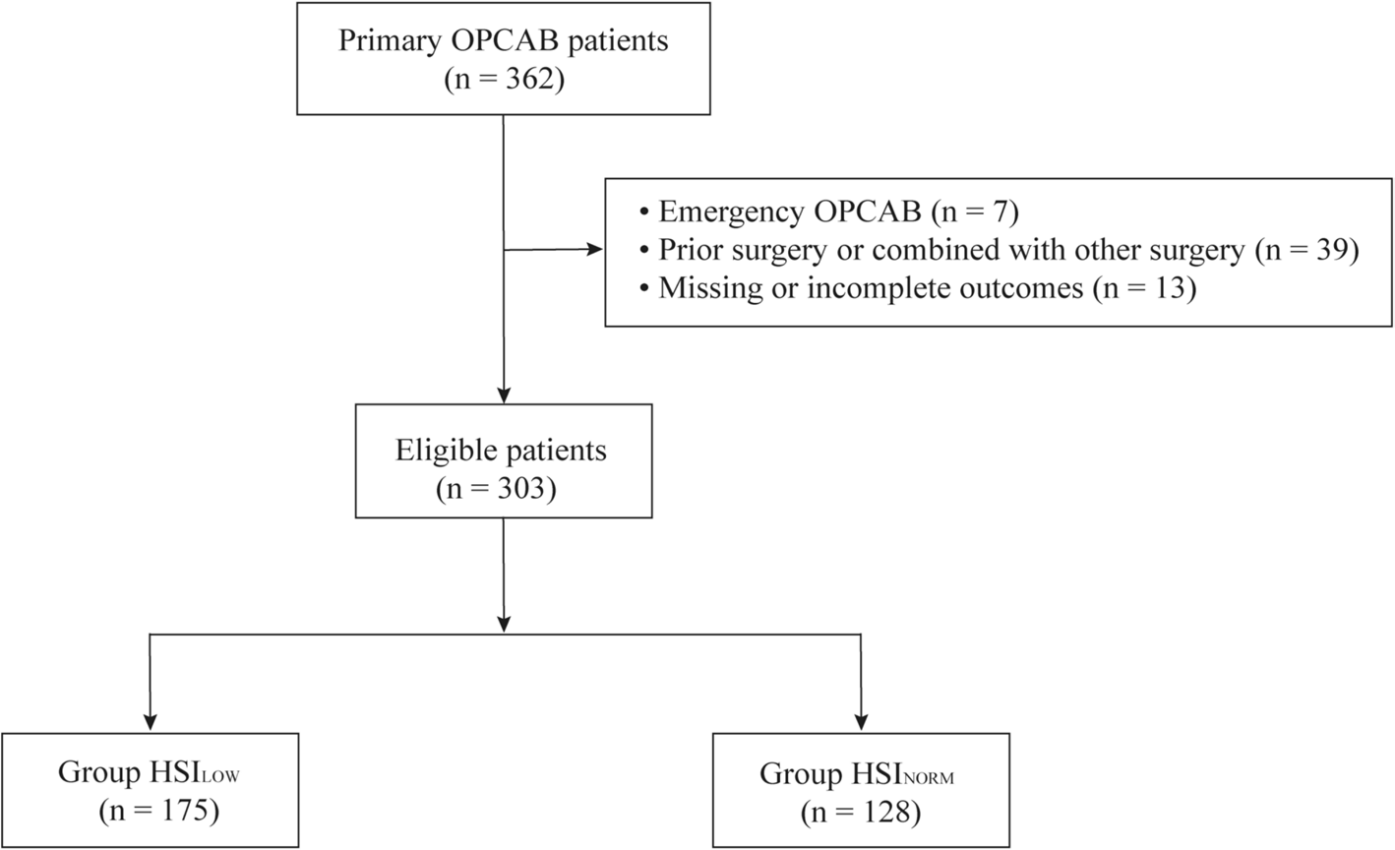
Flowchart of the study. OPCAB, off-pump coronary artery bypass grafting; HSI_LOW_, HSI value < 1.3; HSI_NORM_, HSI value ≥ 1.3

From March 2021 to July 2022, 362 patients underwent elective OPCAB, and this retrospective analysis ultimately included 303 cases (Fig. 1). Among them, 175 (57.76%) patients were classified into HSI_LOW_ group, whereas the remaining 128 (42.24%) patients were assigned to HSI_NORM_ group.

### Characteristics of the patients and preoperative parameters

Table 1 showed the characteristics of the enrolled patients. HSI_LOW_ group had higher preoperative PLT count (221 × 10^9^/L *vs*. 202 × 10^9^/L; *P* = 0.041) and platelet crit (PCT) value (0.23% *vs*. 0.22%; *P* = 0.040) compared to HSI_NORM_ group.

**Table 1:** Characteristics of enrolled patients [median (IQR) or cases (%)]

Variables	HSI_LOW_ (*n* = 175)	HSI_NORM_ (*n* = 128)	*P* value
Age (years)	63.00 (55.50, 68.00)	62.50 (56.00, 67.25)	0.930
Male (%)	141 (80.6)	96 (75.0)	0.308
BMI (kg/m^2^)	25.80 (22.49, 29.01)	26.87 (23.02, 30.81)	0.107
Smoker (%)	86 (49.1)	69 (53.9)	0.413
Hypertension (%)	112 (64.0)	85 (66.4)	0.755
Diabetes mellitus (%)	72 (41.1)	53 (41.4)	1.000
Hyperlipidemia (%)	147 (84.0)	116 (90.6)	0.131
COPD (%)	3 (1.7)	0 (0.0)	0.367
Neurological events (%)	31 (17.7)	23 (18.0)	1.000
LVEF (%)	61.00 (57.00, 65.00)	62.00 (58.00, 65.00)	0.229
AF (%)	5 (2.9)	3 (2.3)	1.000
Prior MI (%)	0 (0.0)	0 (0.0)	NA
Prior PH (%)	2 (1.1)	0 (0.0)	0.620
Statins (%)	165 (94.3)	122 (95.3)	0.693
β-blockers (%)	137 (78.3)	104 (81.2)	0.626
ARB (%)	46 (26.3)	38 (29.7)	0.513
ACEI (%)	14 (8.0)	10 (7.9)	0.968
Nitrates (%)	159 (90.9)	118 (92.2)	0.841
Preoperative use of clopidogrel (%)	3 (1.7)	6 (4.7)	0.515
Preoperative use of aspirin (%)	12 (6.9)	13 (10.2)	0.781
Preoperative use of LMWH (days)	4.00 (2.00, 5.00)	4.00 (2.00, 6.00)	0.245
Blood type A (%)	54(30.9)	35(27.3)	0.507
Blood type B (%)	64(36.6)	47(36.7)	0.979
Blood type AB (%)	13(7.4)	17(13.3)	0.092
Blood type O (%)	44(25.1)	29(22.7)	0.617
Hemoglobin (g/L)	138.00 (126.00, 147.00)	135.00 (125.00, 147.00)	0.375
PLT count (10^9^/L)	221.00 (179.50, 258.50)	202.00 (169.25, 253.00)	0.041
Platelet distribution width (fL)	12.20 (11.10, 13.15)	12.00 (11.30, 13.40)	0.466
Mean platelet volume (fL)	10.60 (10.00, 11.10)	10.50 (9.88, 11.13)	0.437
Platelet-large cell rate (%)	29.00 (24.30, 33.00)	29.20 (25.00, 34.00)	0.435
PCT (%)	0.23 (0.20, 0.27)	0.22 (0.18, 0.26)	0.040
PT (seconds)	13.00 (12.55, 13.40)	12.95 (12.58, 13.50)	0.935
INR (ratio)	0.97 (0.94, 1.02)	0.98 (0.94, 1.01)	0.913
APTT (seconds)	35.70 (33.45, 38.45)	35.30 (32.50, 37.60)	0.201
Fibrinogen (g/L)	3.35 (2.91, 3.87)	3.35 (3.04, 3.82)	0.599
D-dimer assay (mg/L)	0.25 (0.21, 0.39)	0.27 (0.21, 0.39)	0.543
FDP (mg/L)	2.50 (2.50, 2.50)	2.50 (2.50, 2.50)	0.459
PAg-AA (%)	15.97 (7.00, 77.58)	27.18 (7.86, 76.21)	0.823
PAg-ADP (%)	59.30 (40.02, 72.98)	56.25 (39.10, 70.97)	0.322
PAg-ADR (%)	73.59 (26.76, 84.92)	55.56 (41.78, 72.92)	0.643
PAg-COL (%)	77.04 (41.34, 85.00)	69.27 (61.08, 74.85)	0.440
Protein C (%)	109.00 (94.50, 118.50)	102.00 (89.25, 121.00)	0.633
Protein S (%)	98.10 (82.70, 119.15)	98.60 (76.03, 127.50)	0.861
Antithrombin III (%)	98.00 (86.50, 109.00)	99.50 (87.50, 107.00)	0.611
vWF: Ag (%)	181.75 (134.58, 214.22)	148.00 (124.58, 195.87)	0.278
Hypersensitive troponin (ng/mL)	0.007 (0.003, 0.022)	0.007 (0.004, 0.012)	0.813
Myoglobin (ng/mL)	33.68 (25.75, 44.04)	35.79 (23.95, 41.72)	0.760
CK (ng/mL)	57.00 (43.25, 83.75)	61.00 (43.00, 83.00)	0.843
CK-MB (ng/mL)	0.92 (0.63, 1.32)	0.88 (0.66, 1.19)	0.293
NTpro-BNP (pg/mL)	219.00 (74.00, 517.00)	209.00 (80.18, 481.00)	0.855
Albumin (g/L)	39.80 (37.35, 42.15)	40.10 (38.00, 43.00)	0.059
ALT (U/L)	20.00 (13.50, 28.50)	23.50 (15.75, 34.25)	0.730
AST (U/L)	25.00 (20.00, 29.50)	26.00 (22.00, 33.00)	0.445
Creatinine (μmol/L)	82.46 (71.50, 97.08)	84.36 (74.23, 95.72)	0.588
Cystatin C (mg/L)	0.94 (0.77, 1.06)	0.93 (0.81, 1.07)	0.193
hs-CRP (mg/L)	0.87 (0.52, 2.57)	1.02 (0.44, 1.84)	0.388
CRP (mg/L)	2.36 (1.74, 4.31)	2.38 (1.63, 3.54)	0.183

*ACEI* Angiotensin-converting enzyme inhibitors, *AF* Atrial fibrillation, *ALT* Alanine transaminase, *ARB* Angiotensin receptor inhibitors, *APTT* Activated partial thromboplastin time, *AST* Aspartate aminotransferase, *BMI* Body mass index, *CK* Creatine kinase, *CK-MB* Creatine kinase isoenzyme, *COPD* Chronic obstructive pulmonary disease, *CRP* C-reactive protein, *FDP* Fibrinogen degradation products, *hs-CRP* Hypersensitive C-reactive protein, *INR* International normalized ratio, *IQR* Interquartile range, *LMWH* Low-molecular-weight heparin, *LVEF* Left ventricular ejection fraction, *MI* Myocardial infarction, *NA* Not applicable, *Neurological* *events* Cerebral stroke or transient ischemic attack, *NT pro-BNP* Amino-terminal pro-brain natriuretic peptide, *PAg-AA* Platelet aggregation test-arachidonic acid, *PAg-ADP* Platelet aggregation test-adenosine diphosphate, *PAg-ADR* Platelet aggregation test-adrenalin, *PAg-COL* Platelet aggregation test-adrenalin, *PCT* Platelet crit, *PH* Pulmonary hypertension, *PLT* Platelet, *PT* Prothrombin time, *vWF* von Willebrand factor

### Intraoperative parameters

Intraoperative parameters were summarized in Table 2. The difference in baseline ACT value (111.95 ± 10.88 s *vs*. 110.43 ± 10.19 s; *P* = 0.233) and ACT value after protamine neutralization (121.29 ± 11.95 s *vs*. 122.14 ± 13.34 s; *P* = 0.624) between HSI_LOW_ group and HSI_NORM_ group were not statistically significant. However, ACT value after initial UFH administration in HSI_NORM_ group was higher than that in HSI_LOW_ group [73.64 s, 95% confidence interval (CI): -79.28, -68.01; *P* < 0.001). The dose of protamine in HSI_LOW_ patients was less than that in HSI_NORM_ group (150 mg *vs.* 160 mg; *P* = 0.005), but the two groups showed no significant differences in dose of UFH (21,300 IU *vs.* 22,200 IU; *P* = 0.158) and protamine/UFH dose ratio (0.67 *vs.* 0.68; *P* = 0.287).

**Table 2:** Intraoperative parameters [median (IQR), cases (%), or x̄ ± s]

Variables	HSI_LOW_ (*n* = 175)	HSI_NORM_ (*n* = 128)	*P* value
ACT value			
baseline ACT	111.95 ± 10.88	110.43 ± 10.19	0.233
ACT after initial UFH administration	326.84 ± 39.80	400.48 ± 34.38	0.000
ACT after protamine neutralization	121.29 ± 11.95	122.14 ± 13.34	0.624
Surgery duration (minutes)	207 (178, 230)	205.00 (179, 231)	0.780
Infused fluid volume (mL)	1,900 (1,500, 2,200)	2,000 (1,700, 2,500)	0.730
Pulmonary artery catheter insertion (%)	100 (57.1)	77 (60.6)	0.625
UFH (IU)	21,300 (17,700, 24,600)	22,200 (18,000, 25,800)	0.158
Protamine (mg)	150 (130, 170)	160 (140, 180)	0.005
Protamine/UFH dose	0.67 (0.65, 0.77)	0.68 (0.65, 0.82)	0.287

*ACT* Activated clotting time, *IQR* Interquartile range, *UFH* Unfractionated heparin

### Postoperative parameters and complications

Postoperative parameters were shown in Table 3. The two groups showed no significant differences in terms of postoperative blood loss at 24 h (460 mL *vs.* 470 mL; *P* = 0.252), total blood loss (920 mL *vs.* 980 mL; *P* = 0.063), and transfusion requirement of RBC (3.4% *vs.* 3.1%; *P* = 1.000) and FFP (3.4% *vs.* 6.2%; *P* = 0.380). In addition, HSI_LOW_ and HSI_NORM_ groups showed no significant differences in terms of postoperative mechanical ventilation duration (MVD) (16 h *vs.* 16 h; *P* = 0.776), chest drainage duration (4 days *vs.* 4 days; *P* = 0.757), ICU LOS (46 h *vs.* 45 h; *P* = 0.114), hospital stay (7 days *vs.* 7 days; *P* = 0.329), and other complications. No patient in either group required PC transfusion or died during the postoperative hospitalization period. In thrombelastography, HSI_LOW_ group showed a shorter kinetics time (2.20 min *vs.* 2.50 min; *P* = 0.010) and a larger alpha angle (58.50° *vs.* 54.10°, *P* = 0.003) than HSI_NORM_ group.

**Table 3:** Postoperative parameters [median (IQR) or cases (%)]

Variables		HSI_LOW_ (*n* = 175)	HSI_NORM_ (*n* = 128)	*P* value
Postoperative blood loss at 24 h (mL)		460 (350, 600)	470 (380, 640)	0.252
Total postoperative blood loss (mL)		920 (685, 1235)	980 (817, 1250)	0.063
RBC transfusion rate (%)		6 (3.4)	4 (3.1)	1.000
FFP transfusion rate (%)		6 (3.4)	8 (6.2)	0.380
PC transfusion rate (%)		0 (0.0)	0 (0.0)	NA
RBC transfusion volume (unit)		2 (2, 2)	2 (2, 2)	0.841
FFP transfusion volume (mL)		400 (0, 400)	400 (400, 400)	0.143
PC transfusion volume (mL)		0 (0, 0)	0 (0, 0)	NA
MVD (hours)		16.00 (13.00, 18.00)	16.00 (13.00, 18.00)	0.776
Chest drainage duration (days)		4.00 (4.00, 5.00)	4.00 (4.00, 5.00)	0.757
LOS in the ICU (hours)		46.00 (23.00, 93.00)	45.00 (22.00, 71.00)	0.086
LOS in the hospital (days)		7.00 (6.00, 8.00)	7.00 (6.00, 8.00)	0.329
Mortality (%)		0 (0.0)	0 (0.0)	NA
Reoperation (%)		3 (1.7)	4 (3.1)	0.674
Cardiac arrest (%)		0 (0.0)	0 (0.0)	NA
MI (%)		0 (0.0)	0 (0.0)	NA
IABP (%)		2 (1.1)	0 (0.0)	0.225
AF (%)		2 (1.1)	2 (1.6)	1.000
New pacemaker (%)		0 (0.0)	0 (0.0)	NA
Neurological events (%)		0 (0.0)	1 (0.8)	0.875
Delirium (%)		2 (1.1)	1 (0.8)	1.000
Renal replacement therapy (%)		0 (0.0)	0 (0.0)	NA
Dopamine (%)		157 (89.7)	115 (89.8)	1.000
Nitroglycerin (%)		118 (67.4)	81 (63.3)	0.530
Norepinephrine (%)		64 (36.6)	52 (40.6)	0.550
Thromboelastography	reaction time (minutes)	7.10 (6.10, 8.40)	7.55 (6.38, 8.35)	0.134
	kinetics time (minutes)	2.20 (1.80, 2.60)	2.50 (1.90, 2.92)	0.010
	Alpha angle (degrees)	58.50 (53.35, 63.25)	54.10 (48.88, 60.20)	0.003
	maximum amplitude (millimeters)	55.60 (50.60, 61.20)	53.85 (48.97, 60.20)	0.195
	lysis at 30 min (%)	0.10 (0.10, 0.20)	0.10 (0.10, 0.30)	0.195
Blood coagulation factors	II (%)	82.90 (78.10, 88.10)	93.10 (76.90, 100.45)	0.249
	V (%)	106.00 (96.15, 121.80)	108.70 (105.95, 116.00)	0.311
	VII (%)	71.80 (60.85, 83.57)	71.80 (65.55, 85.50)	0.639
	X (%)	82.35 (67.85, 90.33)	82.10 (75.80, 95.70)	0.888
	VIII (%)	122.20 (100.38, 149.15)	121.10 (102.80, 145.00)	0.961
	IX (%)	114.10 (107.53, 138.67)	107.60 (97.55, 129.50)	0.336
	XI (%)	105.20 (85.00, 119.40)	90.20 (85.80, 100.50)	0.269
	XII (%)	74.30 (62.23, 92.17)	60.40 (53.00, 85.50)	0.183
Big-endothelin (pmol/L)		0.42 (0.31, 0.56)	0.46 (0.37, 0.61)	0.379
IL-1β (pg/mL)		1.69 (1.12, 2.24)	1.59 (0.99, 2.33)	0.589
IL-2 (pg/mL)		1.54 (1.31, 1.96)	1.56 (1.31, 2.08)	0.588
IL-4 (pg/mL)		1.54 (1.00, 2.04)	1.50 (1.05, 2.12)	0.831
IL-5 (pg/mL)		1.38 (1.14, 1.86)	1.27 (1.09, 1.65)	0.261
IL-6 (pg/mL)		134.87 (82.65, 202.58)	135.43 (84.20, 234.44)	0.445
IL-8 (pg/mL)		40.00 (24.90, 58.00)	44.29 (31.69, 62.92)	0.167
IL-10 (pg/mL)		9.75 (5.88, 16.08)	10.93 (6.62, 19.39)	0.160
IL-12 (pg/mL)		1.86 (1.10, 2.71)	1.92 (1.00, 2.83)	0.724
IL-17 (pg/mL)		2.73 (0.01, 6.04)	3.24 (0.37, 5.68)	0.750
TNF-a (pg/mL)		1.78 (1.18, 2.99)	1.66 (1.01, 2.66)	0.233
IFN-a (pg/mL)		1.67 (1.16, 2.26)	1.67 (1.19, 2.22)	0.724
IFN-γ (pg/mL)		1.44 (1.07, 1.93)	1.48 (1.04, 1.93)	0.954

*AF* Atrial fibrillation, *FFP* Fresh frozen plasma, *IABP* Intra-aortic balloon pump, *ICU* Intensive care unit, *IFN* Interferon, *IL* Interleukin, *IQR* Interquartile range, *LOS* Length of stay, *MI* myocardial infarction, *MVD* Mechanical ventilation duration, *NA* Not applicable, *Neurological events* Cerebral stroke or transient ischemic attack, *PC* Platelet concentrates, *RBC* Red blood cell, *TNF* Tumor necrosis factor

### Multivariable logistic regression analysis

Ten variables were included in multivariate logistic regression analysis (Table 4). Age, sex, and duration of preoperative LMWH use were considered clinically relevant variables [12–15]. The other seven variables showed *P* < 0.20 in the univariate analysis. Although the proportion of blood type AB among preoperative patients showed *P* < 0.20 in the univariate analysis, owing to the limited number of available events, this factor was not included in the multivariate logistic regression analysis. High preoperative PLT count (odds ratio: 1.006; 95% CI: 1.002, 1.011; *P* = 0.008) was significantly associated with low intraoperative HSI value.

**Table 4:** Multivariate logistic regression analysis for heparin sensitivity index

Predictors	B	OR (95% CI)	*P* value
Age	0.016	1.015 (0.702, 2.547)	0.296
Male	0.274	1.337 (0.702, 2.547)	0.377
BMI	– 0.042	0.959 (0.916, 1.005)	0.080
Hyperlipidemia	– 0.509	0.601 (0.277, 1.302)	0.197
PLT count	0.006	1.006 (1.002, 1.011)	0.008
PCT	–0.196	0.820 (0.393, 1.713)	0.598
Albumin	– 0.026	0.973 (0.926, 1.022)	0.271
Cystatin C	– 0.204	0.809 (0.419, 1.562)	0.528
CRP	0.069	1.069 (0.992, 1.152)	0.790
LMWH	– 0.039	0.967 (0.877, 1.066)	0.497

*B* Regression coefficient, *BMI* Body mass index, *CI* Confidence interval, *CRP* C-reactive protein, *LMWH* Low-molecular-weight heparin, *OR* Odds ratio, *PCT* Platelet crit, *PLT* platelet

## Discussion

This study showed no increase in postoperative blood loss at 24 h in Chinese patients with a low HSI undergoing elective OPCAB. In the assessment of risk factors for low intraoperative HSI value, preoperative high PLT count was identified as an independent predictor.

The low intraoperative HSI value is associated with concerns regarding potential overactivation of the clotting system due to inadequate anticoagulation, necessitating the administration of additional UFH. However, this exposes surgical patients to the risk of excessive UFH use [8]. Furthermore, repeated administration of UFH at doses exceeding the predicted range may necessitate increased protamine administration to counteract the effects of UFH, thereby exposing patients to the risk of protamine overdose [9]. An excess of protamine can attenuate thrombin-induced PLT aggregation and reduce the responsiveness of PLT to thrombin receptor agonist peptides. Additionally, excessive protamine administration weakens clot strength and shortens the time for clot lysis while promoting fibrinolysis mediated by tissue-type plasminogen activators, thereby facilitating clot breakdown [16]. These factors may contribute to increased postoperative blood loss. In this study, although the ACT value after initial UFH administration in HSI_LOW_ group was lower than that in HSI_NORM_ group, there was no increase in blood loss at 24 h or transfusion requirement was observed after OPCAB in HSI_LOW_ group. The plausible reasons for these findings are as follows: the use of UFH in HSI_LOW_ group was not greater than that in HSI_NORM_ group. Similarly, the protamine/UFH dose ratio in both groups ranged from 0.6 to 0.7 and did not differ significantly between the two groups. The reaction time of the TEG after surgery in both groups, which indicated the onset of measurable clot strength, showed no residual effect of UFH. Some studies have demonstrated a half-dose of protamine can safely reverse UFH without increasing postoperative blood loss [17, 18]. In this regard, a protamine/UFH dose ratio ranging from 0.6 to 0.7 is considered an appropriate neutralization ratio.

Another crucial factor that cannot be overlooked is the perioperative coagulation profile of OPCAB, which exhibits a lower degree of coagulation-fibrinolysis activation and PLT dysfunction [19, 20]. Moreover, in comparison with on-pump cardiac surgery, OPCAB circumvents the impact of hemodilution and hypothermia on the coagulation system [8]. The aforementioned factors collectively contribute to the development of a procoagulant state following OPCAB. Postoperative TEG revealed coagulation characteristics, indicating accelerated blood clot formation, as evidenced by the K value in both groups falling below the lower limit of normal (4 min–9 min). The K value was shorter and the alpha angle was larger in HSI_LOW_ group than in HSI_NORM_ group, indicating higher procoagulant activity reflective of increased fibrinogen function. Previous studies have highlighted potential thromboembolic risks associated with reduced heparin responsiveness [7]. Inadequate response to UFH may result in intraoperative and postoperative coagulation within the peripheral vascular system [20], thereby increasing the risk of endangering coronary anastomosis patency [21]. Despite the strong theoretical possibility of a close relationship between reduced heparin responsiveness and postoperative MI in patients undergoing OPCAB [9], no association between low intraoperative HSI value and adverse ischemic outcomes was observed in this study. The etiology of postoperative MI is multifactorial. In addition to the hypercoagulable state, surgical techniques are also considered significant contributing factors. The present study involved ten experienced faculty surgeons who had completed at least 100 OPCAB procedures as part of their institutional requirement before being qualified to perform the procedure [22]. Therefore, the quality of surgery in this study was deemed reliable.

The present study also confirmed the correlation between preoperative high PLT count and low intraoperative HSI value [12, 13, 23]. The reduced responsiveness to UFH was attributed to excessive production of platelet factor 4 (PF4), an anti-heparin agent, resulting from the accelerated rate of PLT destruction. The strong binding affinity of PF4 for UFH leads to the displacement of PF4 from vascular binding sites with lower affinity, resulting in the formation of circulating complexes with UFH that neutralize its effect. Therefore, one possible explanation for low intraoperative HSI value in patients with preoperative high PLT count may be their greater capacity to produce PF4 after PLT activation [12]. Additionally, PLT count was associated with ACT value [13, 23], which are currently regarded as the standard parameter for monitoring the anticoagulant effect of UFH. The measurement of ACT is a test of whole-blood coagulation that involves the interaction between plasma coagulation components and PLT phospholipids in the hemostatic process. Therefore, a high PLT count may shorten the ACT response time.

In this study, preoperative LMWH treatment was not associated with the incidence of low intraoperative HSI value. Some studies have demonstrated improved outcomes with LMWH use in patients with unstable angina [6]. Therefore, all patients presenting for OPCAB received LMWH before surgery. The dose of enoxaparin was 6000 IU, administered subcutaneously every 12 h. Preoperative heparin exposure was equivalent between the two groups.

Preoperative administration of antiplatelet agents, such as aspirin and clopidogrel, has been linked to an increased risk of blood loss after cardiac surgery [11, 24]. According to the European guidelines [25], for clopidogrel, from the perspective of reducing the blood loss risk, elective cardiac surgery should be performed five days after stopping clopidogrel. The European guidelines also recommend that patients at low risk of perioperative blood loss should continue taking aspirin and do not need to stop taking aspirin before surgery. However, one study indicated that the preoperative use of aspirin significantly increases the risk of postoperative blood loss and blood transfusion in patients undergoing OPCAB [11], and recommended discontinuing aspirin intake six days prior to surgery. In this study, preoperative use of clopidogrel or aspirin was not included in the multivariate logistic regression model because of the limited number of available events. Further research is necessary to investigate whether clopidogrel or aspirin increases the risk of postoperative blood loss during OPCAB.

Limitations of the study include the following. First, this study had a retrospective design. The results of the current study rely heavily on the comprehensiveness and accuracy of the original documentation. We endeavored to minimize potential inaccuracies by meticulously reviewing patients’ medical records. Secondly, HSI was used as a criterion to indicate heparin responsiveness, with some studies using a threshold value of 1.3 and others using 1.0. A stringent criterion for low intraoperative HSI value was defined as an HSI value less than 1.0, which showed an incidence rate between 15.5% [26] and 20.8% [2]. Despite the low incidence of low intraoperative HSI value as measured by this stringent criteria, future studies involving large samples should pay greater attention to this issue.

## Conclusions

The present study demonstrated that intraoperative HSI was not associated with postoperative blood loss in Chinese patients undergoing elective OPCAB. Preoperative high PLT count was an independent predictor of low intraoperative HSI value.

## Data Availability

The datasets used and analyzed during the current study are available from the corresponding author on reasonable request.

## Abbreviations

ACT: Activated clotting time
AF: Atrial fibrillation
BIS: Bispectral index
FFP: Fresh frozen plasma
HSI: Heparin sensitivity index
IABP: Intra-aortic balloon pump
ICU: Intensive care unit
LMWH: Low-molecular-weight heparin
LOS: Length of stay
MI: Myocardial infarction
OPCAB: Off-pump coronary artery bypass grafting
PC: Platelet concentrates
PCT: Platelet crit
PF4: Platelet factor 4
PLT: Platelet
RBC: Red blood cell
TEG: Thromboelastography
UFH: Unfractionated heparin
